# Cuban policosanol improves high-density lipoprotein cholesterol efflux capacity in healthy Japanese subjects

**DOI:** 10.3389/fnut.2023.1297008

**Published:** 2024-01-08

**Authors:** Yoshinari Uehara, Tomohiro Komatsu, Kei Sasaki, Satomi Abe, Shihoko Nakashima, Taiki Yamamoto, Ji-Eun Kim, Kyung-Hyun Cho

**Affiliations:** ^1^Faculty of Sports and Health Science, Fukuoka University, Fukuoka, Japan; ^2^Research Institute for Physical Activity, Fukuoka University, Fukuoka, Japan; ^3^Center for Preventive, Anti-aging and Regenerative Medicine, Fukuoka University Hospital, Fukuoka, Japan; ^4^Raydel Research Institute, Medical Innovation Complex, Daegu, Republic of Korea; ^5^LipoLab, Yeungnam University, Gyeongsan, Republic of Korea

**Keywords:** policosanol, high-density lipoprotein, HDL cholesterol efflux capacity, apolipoprotein A-I, lipid metabolism

## Abstract

Policosanol supplementation has been reported to increase high-density lipoprotein (HDL)-cholesterol (HDL-C). However, the association between Cuban policosanol supplementation and HDL cholesterol efflux capacity (CEC), an important function of HDL, remains unclear. We performed a lipoprotein analysis investigating 32 Japanese healthy participants (placebo, *n* = 17 or policosanol supplementation for 12 weeks, *n* = 15) from a randomized Cuban policosanol clinical trial. First, HDL CEC and HDL-related factors were measured before and after policosanol supplementation. Then, through electron microscopy after ultracentrifugation and high-performance liquid chromatography, HDL morphology and subclass were analyzed, respectively. Finally, the effects of policosanol supplementation regarding HDL function, HDL-related factors, and HDL morphology/component were examined. Cuban policosanol considerably increased the HDL CEC and HDL-C and apolipoprotein A-I (ApoA-I) levels. Furthermore, policosanol supplementation led to larger HDL particles, increased cholesterol content in larger HDL particles, and reduced triglyceride content in smaller HDL particles. In participants with high baseline HDL-C levels, the policosanol effects for HDL CEC are observed. HDL CEC fluctuation induced by policosanol was highly associated with HDL-C and ApoA-I changes. In conclusion, for the first time, we demonstrated that policosanol supplementation increased the HDL CEC in healthy participants.

## 1 Introduction

A low high-density lipoprotein (HDL)-cholesterol (HDL-C) level is widely recognized to be associated with mortality and atherosclerotic cardiovascular disease (ASCVD) according to findings from previous epidemiological studies conducted worldwide (1–3) and in Japan (4). Recently, the Mendelian randomization study, which is a study method reflecting gene background effects from birth to death in humans, revealed that some HDL-C lowering genes were not associated with ASCVD occurrence (5, 6). Conversely, the same study also revealed that a low HDL-C concentration was correlated with ASCVD in the group analysis excluding those with gene mutation backgrounds (5). Thus, secondary factors may be more crucial than the genetic background in reducing HDL-C concentration associated with ASCVD.

HDL possesses multi-functional anti-atherosclerotic effects, such as anti-inflammation, apoptotic inhibition, anti-oxidation, and anti-thrombotic actions (7). HDL cholesterol efflux capacity (CEC), one of the primary HDL functions, extracts excess cholesterol from the atherosclerotic plaque and is the first step in the process of reverse cholesterol transport (RCT), which is an atheroprotective mechanism (8). Then, HDL in the circulating blood transports excess cholesterol from the peripheral tissues to the liver, and the cholesterol is eliminated outside of the body via bile and feces, which is referred to as the RCT process (9). Apolipoprotein A-I (ApoA-I), a major HDL protein, is an essential biogenesis factor of HDL via maturation of binding phospholipids and free cholesterol in the ATP-binding cassette transporter (ABC) A1-mediated process from the cell membrane (10). Several human clinical trials reveal that HDL CEC is a powerful indicator of cardiovascular disease (CVD), independent of HDL-C (7, 11). HDL CEC has been also recognized to be valuable and useful for CVD prognosis among Japanese individuals (12). According to the meta-analysis findings elucidating that HDL CEC is inversely related to CVD risk (13), HDL CEC elevation is expected to have beneficial effects on CVD and mortality risks.

Policosanol has been recommended by the American Heart Association Scientific Statement as one of the alternative medicines with optimal effects on heart disease (14). In prior reports (15, 16), Cuban policosanol is defined as a mixture of aliphatic alcohols ranging from 24 to 34 carbon atoms, among many kinds of policosanol purified from various plant sources. The previous study reported that octacosanol, one of the main Cuban policosanol, was largely distributed in the liver, adipose tissue, and muscle after administration in an animal model (17). Other several animal studies of hyperlipidemic mice demonstrated that policosanol had the effects of decreasing lipid accumulation in the liver through bile acid (18), decreasing fatty acid synthesis in white adipose tissue, and increasing uncoupling protein-1 (19)/peroxisome proliferator-activated receptor gamma coactivator-1a in brown adipose tissue (20). However, currently, policosanol metabolism in the body remains poorly elucidated. Previous human studies showed that policosanol has nutraceutical benefits for hypertension (21) and dyslipidemia (22) management. For lipid modification, policosanol reduces low-density lipoprotein cholesterol (LDL-C) and elevates HDL-C concentrations (22). Although the LDL-C reduction effects by policosanol is recognized as similar to a mild statin (23), strong statins, which have a more powerful action, are reported to have superior effects for LDL-C reduction than policosanol (24). However, a prior study demonstrated that policosanol increased the HDL-C levels more than that of a statin. Similarly, Cuban policosanol is reported to have a similar effect of elevating HDL-C concentrations (15). However, policosanol effects on HDL, especially on HDL function, remains poorly explored. Thus, in the current study, we aimed to investigate the following factors via a lipoprotein analysis of a Cuban policosanol clinical trial in Japanese healthy participants: (1) whether Cuban policosanol influenced HDL function, morphology, and lipid content, (2) who will likely benefit from the policosanol effect in HDL among healthy participants, and (3) what HDL component factors are associated with HDL function.

## 2 Materials and methods

### 2.1 Study design and population

The present study is a lipoprotein analysis from a previous double-blind, randomized, and placebo-controlled trial that utilized policosanol (Raydel^®^ Cuban Policosanol, 20 mg/day) or a placebo that was taken for 12 weeks by healthy Japanese participants (15). Cuban policosanol was defined as a genuine policosanol with a specific ratio of each ingredient (16): 1-tetracosanol (C24H49OH, 0.1–20 mg/g); 1-hexacosanol (C26H53OH, 30.0–100.0 mg/g); 1-heptacosanol (C27H55OH, 1.0–30.0 mg/g); 1-octacosanol (C28H57OH, 600.0–700.0 mg/g); 1-nonacosanol (C29H59OH, 1.0–20.0 mg/g); 1-triacontanol (C30H61OH, 100.0–150.0); 1-dotriacontanol (C32H65OH, 50.0–100.0 mg/g); and 1-tetratriacontanol (C34H69OH, 1.0–50.0 mg/g). Briefly, the participants were stratified into the policosanol or placebo groups and took two tablets containing policosanol 10 mg (Raydel^®^) or a placebo per day. The tablet in the policosanol group included policosanol (10 mg), hydroxypropyl cellulose, carboxymethyl cellulose, maltodextrin, lactose, and crystalline cellulose. In the placebo group, the tablet contained maltodextrin (10 mg), rather than policosanol. The Raydel^®^ policosanol tablet was acquired from Raydel Japan (Tokyo, Japan), which was manufactured with Cuban policosanol at Raydel Australia (Thornleigh, Sydney). According to the exclusion criteria described below, the placebo group comprised 17 participants (male = 9, female = 8), whereas the policosanol group included 15 participants (male = 8, female = 7).

In the present study, the inclusion criteria were the same as those described in the main randomized clinical trial (15). All participants were advised to refrain from ingesting excess food/cholesterol and alcohol drinking. Moreover, before the study and during the study period, smoking both directly and indirectly was not allowed. The inclusion criteria were as follows: age between 20 and 65 years and serum LDL-C levels in the normal and mildly elevated range (120–159 mg/dL). In the current study, the exclusion criteria were more strict in terms of dietary habits, including food and drink, in each self-questionnaire to eliminate the impact on lipid profile, as referenced from the Japan Atherosclerosis Society Guidelines for the Prevention of Atherosclerotic Cardiovascular Diseases 2017 (25). The exclusion criteria were as follows: (1) maintenance treatment for metabolic disorders, including dyslipidemia, hypertension, and diabetes; (2) severe hepatic, renal, cardiac, respiratory, endocrinological, and metabolic diseases; (3) allergies; (4) heavy drinkers, consuming >30 g and 15 g of alcohol per day for men and women, respectively; (5) taking medicine or functional food products that may affect lipid metabolism, including an increase in HDL-C concentration or a decrease in LDL-C concentration and a decrease in the triglyceride concentration; (6) current and past smokers; (7) pregnant or lactating women or women planning to become pregnant during the study period; (8) who donated >200 mL of blood within 1 month or 400 mL of blood within 3 months prior to clinical trial initiation; (9) who participated in other clinical trials within the last 3 months or is currently participating in another clinical trial; (10) those who consumed >2,000 kcal per day or cholesterol intake >600 mg per day; and (11) others considered unsuitable for the study as per the principal investigator’s discretion. The ethics committee of the Koseikai Fukuda Internal Medicine Clinic reviewed and approved the study protocol (Osaka, Japan, IRB approval number 15000074, approval date on September 18, 2021), which was conducted in accordance to the ethical guidelines stipulated in the 1975 Declaration of Helsinki. The protocol was also registered with the University Hospital Medical Information Network Clinical Trial Registry (UMIN000046345). All participants provided written informed consent prior to initiation of any study procedure.

### 2.2 Anthropometric, blood, and lipoprotein analyses

According to the methods described in the randomized clinical study, anthropometric measurement and blood samples were collected from the participants (15). The samples were analyzed by BML Inc. (Tokyo, Japan) for lipid, glucose, and inflammatory factors. According to standard protocols, serum lipoproteins were isolated via ultracentrifugation (26), which were then divided as follows: very-low-density lipoprotein (VLDL) (d < 1.019 g/mL), LDL (1.019 < d < 1.063), HDL_2_ (1.063 < d < 1.125), and HDL_3_ (1.125 < d < 1.225). The total cholesterol and triglyceride levels in each lipoprotein were measured using commercially available kits (Wako Pure Chemical, Osaka, Japan) after lipoprotein purification. Lipoprotein characterization, including the shape and size of the HDL and its particle number, was conducted via transmission electron microscopy (Hitachi H-7800; Ibaraki, Japan) at 40,000× magnification, as described in the previous report (27). Briefly, the HDL size was measured using Image J software version 1.53r^1^ and Hitachi EMIP-EX software (Ver. 07.13, Hitachi Tokyo, Japan) with approximately 70 randomly selected particles. To measure the particle size, manually and randomly selected individual particle image using a tablet pen (CLT-6100WL, Wacom, Saitama, Japan) were calculated using the software. HDL_2_ particle size and diameter (10–15 nm) were larger than that of HDL_3_ (7–9 nm of diameter) using Hitachi EMIP-EX software (Ver. 07.13, Hitachi Tokyo, Japan).

The lipoprotein subclass and composition in plasma, such as chylomicron, VLDL, LDL, and HDL, were analyzed through gel permeation high-performance liquid chromatography (HPLC) using LipoSEARCH (Skylight Biotech, Inc., Akita, Japan) (28, 29). In the HPLC analysis, lipoproteins can be stratified into 20 lipoprotein subclass groups, which are as follows: chylomicron (fractions 1–2), VLDL (fractions 3–7: large, 3–5; medium, 6; small, 7), LDL (fractions 8–13: large, 8; medium, 9; small, 10–13), and HDL (fractions 14–20: large, 14–16; medium, 17; small, 18–20). After the analysis, the cholesterol and triglyceride contents in those subclasses were quantified.

### 2.3 Cell culture and materials

J774A.1 cells (JCRB cell bank, Osaka, Japan) were purchased and cultured in Dulbecco’s Modified Eagle Medium with 4.5 g/L of a high glucose (Sigma-Aldrich, St Louis, MO, United States) medium containing 10% fetal bovine serum (Life Technologies Co, Carlsbad, CA, United States) and 0.5% penicillin/streptomycin.

22(R)-hydroxycholesterol and 9-cis-retinoic acid were purchased from Sigma-Aldrich and LKT Laboratories, Inc. (St Paul, MN, United States), respectively, and were dissolved using a dimethyl sulfoxide solution. Fatty-acid-free bovine serum albumin (BSA) was purchased from Calbiochem (Merck KGaA, Darmstadt, Germany).

### 2.4 HDL cholesterol efflux capacity

The cellular CEC was measured as described previously (30–34). Briefly, J774A.1 cells were radiolabeled with 5 uCi/mL of [1,2-3H] cholesterol (PerkinElmer, Waltham, MA, United States) for 24 h. J774A.1 cells were treated with 5 μmol/L 22(R)-hydroxycholesterol, an LXR agonist, and 2.5 μmol/L 9-cis-retinoic acid, an RXR agonist to activate ABCA1 and ABCG1 for 18 h. Then, 2.0% apolipoprotein B-depleted plasma, which included HDL through ApoB-depleted treatment with the phosphotungstic acid-Mg precipitation method, was added to the cells for 4 h to extract cholesterol from the cells to the supernatant. Finally, the liquid scintillation count was measured, and the efflux rates of 3H-cholesterol from the cells to the medium were analyzed. Serum-free medium containing 0.2% BSA was used as the basis solution for radiolabeled treatment, stimulation, and plasma addition for all experiments on the HDL CEC.

### 2.5 Statistical analysis

Data were expressed as the mean ± standard deviation (SD) or ± standard error of mean, and median with interquartile range as appropriate. The Kolmogorov–Smirnov test for normality was used to evaluate the baseline population characteristics, and the difference was analyzed using an unpaired t-test with normality or Mann–Whitney U test without normality. For triglycerides, apolipoprotein B-48 (ApoB-48), and high sensitivity C-reactive protein (hsCRP), log-transformed values, which were normally distributed, were used to compare them. Using a homogeneity test of the variances through Levene’s statistics for normality, HDL_2_ and HDL_3_ evaluation was performed. Then, a paired *t*-test was performed with normality, and if without normality, nonparametric statistics were performed using the Kruskal–Wallis test, for HDL_2_ and HDL_3_ morphology and composition analysis.

For lipoprotein fractions after normality evaluation, the HPLC data difference was analyzed using an unpaired *t*-test with normality or by Mann–Whitney U test with log-transformed values without normality. In a four-group comparison categorized according to baseline HDL-C levels and sex, differences between groups were analyzed using the Kruskal–Wallis test with Dunn’s multiple comparison test. Using a simple linear regression with Pearson’s product–moment correlation co-efficient with normality or Spearman’s rank correlation coefficient without normality, a single correlation analysis was evaluated. Statistical significance was set at a *p* value < 0.05.

The GraphPad Prism version 8 software (GraphPad software LLC., San Diego, CA, United States) and the JMP Version 12.0 software package (SAS Institute Inc., NC, United States) were used for the statistical analyses, except in the HDL_2_ and HDL_3_ evaluations. In the HDL_2_ and HDL_3_ evaluations, statistical power was estimated using the program G*Power 3.1.9.7 (G*Power from University of Düsseldorf, Düsseldorf, Germany). SPSS software version 29.0 (IBM, Chicago, IL, USA) was used to analyze data.

## 3 Results

### 3.1 Policosanol significantly increased HDL CEC and HDL-C levels

In this paper, lipid profiles and function were analyzed using data from previously published randomized clinical trials [placebo (*n* = 17) or policosanol (20 mg/day, *n* = 15), 12 weeks] (15) to examine the effects and mechanisms of policosanol on lipids. The policosanol effects were investigated through observation of the changes from baseline (week 0) to the end (week 12) of the study in Japanese healthy participants. At baseline, no difference in any factor was found between the placebo and policosanol groups (Table 1).

**Table 1:** Baseline characteristics of the study population.

	Placebo	Policosanol	*p* value
	Total (*n* = 17)	Total (*n* = 15)	
Age, years	54.2 ± 6.5	51.3 ± 7.5	0.241
Male, *n* (%)	9 (53)	8 (53)	
BMI	21.9 ± 2.0	21.0 ± 1.5	0.143
Total cholesterol, mg/dL	222.1 ± 20.8	217.9 ± 14.8	0.521
LDL-cholesterol, mg/dL	133.4 ± 15.4	126.5 ± 10.8	0.159
Triglycerides, mg/dL^a^	74.0 [53.5–86.5]	81.0 [60.0–115.0]	0.118
HDL-cholesterol, mg/dL	66.1 ± 9.9	63.7 ± 11.4	0.532
HDL efflux capacity, %	15.9 ± 0.9	15.5 ± 0.8	0.231
Apolipoprotein AI, mg/dL	174.4 ± 19.2	174.0 ± 19.1	0.959
Apolipoprotein AII, mg/dL	35.5 ± 4.0	33.8 ± 3.2	0.202
Apolipoprotein B100, mg/dL	80.6 ± 12.1	88.0 ± 16.7	0.163
Apolipoprotein B48, mg/dL^a^	0.42 [0.23–0.54]	0.55 [0.21–1.11]	0.527
HbA1c, %	5.5 ± 0.2	5.5 ± 0.3	0.951
Fasting blood glucose, mg/dL	90.4 ± 8.9	91.1 ± 11.7	0.832
hsCRP, mg/L^a^	0.35 [0.20–0.7]	0.21 [0.18–0.52]	0.170

Values are expressed as mean ± standard deviation, except for triglycerides, apolipoprotein B-48, and hsCRP (median [interquartile ranges]).

a log-transformed prior to analysis. BMI, body mass index; LDL, low-density lipoprotein cholesterol; HDL, high-density lipoprotein; HbA1c, hemoglobin A1c; hsCRP, high sensitivity C-reactive protein.

**Figure 1:**
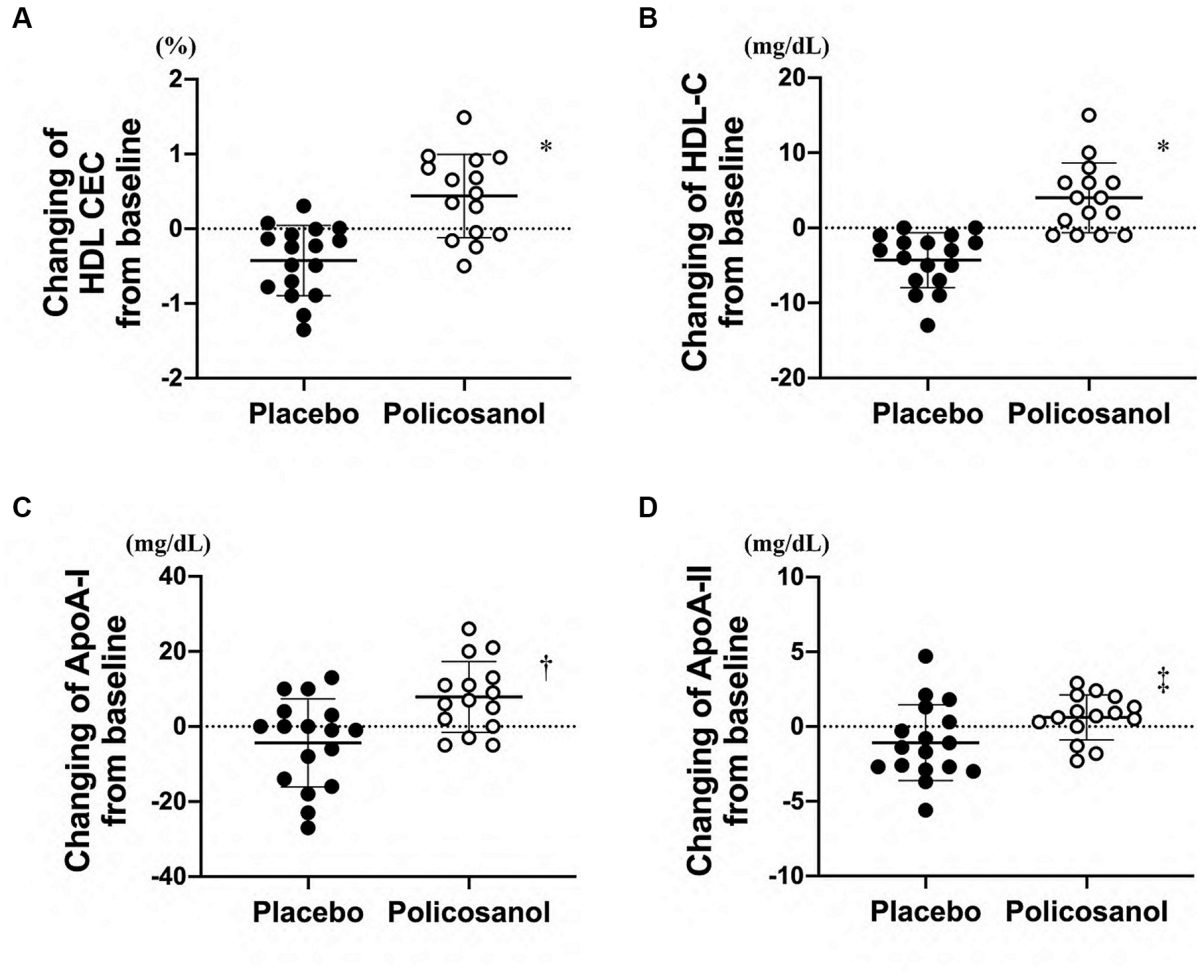
Effects of policosanol supplementation for 12 weeks on HDL-related factors such as **(A)** HDL CEC and **(B)** HDL-C, **(C)** ApoA-I, and **(D)** ApoA-II levels. The analysis was conducted by assessing the differences between the preintervention and postintervention values in the placebo and the policosanol group. Values are expressed as mean ± standard deviation (SD). ^*^p < 0.001; ^†^*p* < 0.01; ^‡^*p* < 0.05 vs. placebo. Placebo group, *n* = 17 and policosanol group, *n* = 15. HDL, high-density lipoprotein; CEC, cholesterol efflux capacity; Apo, apolipoprotein.

The policosanol effects after a 12-week placebo or policosanol supplementation are shown in Figure 1. The HDL CEC upregulation was markedly increased in the policosanol group at week 12, as compared with the placebo group (Figure 1A). Similarly, the HDL-C concentration changes were significantly higher in the policosanol group (Figure 1B). The ApoA-I (Figure 1C) and ApoA-II (Figure 1D) level changes, which are two major HDL constituent proteins, were also significantly increased in the policosanol group at week 12.

**Figure 2:**
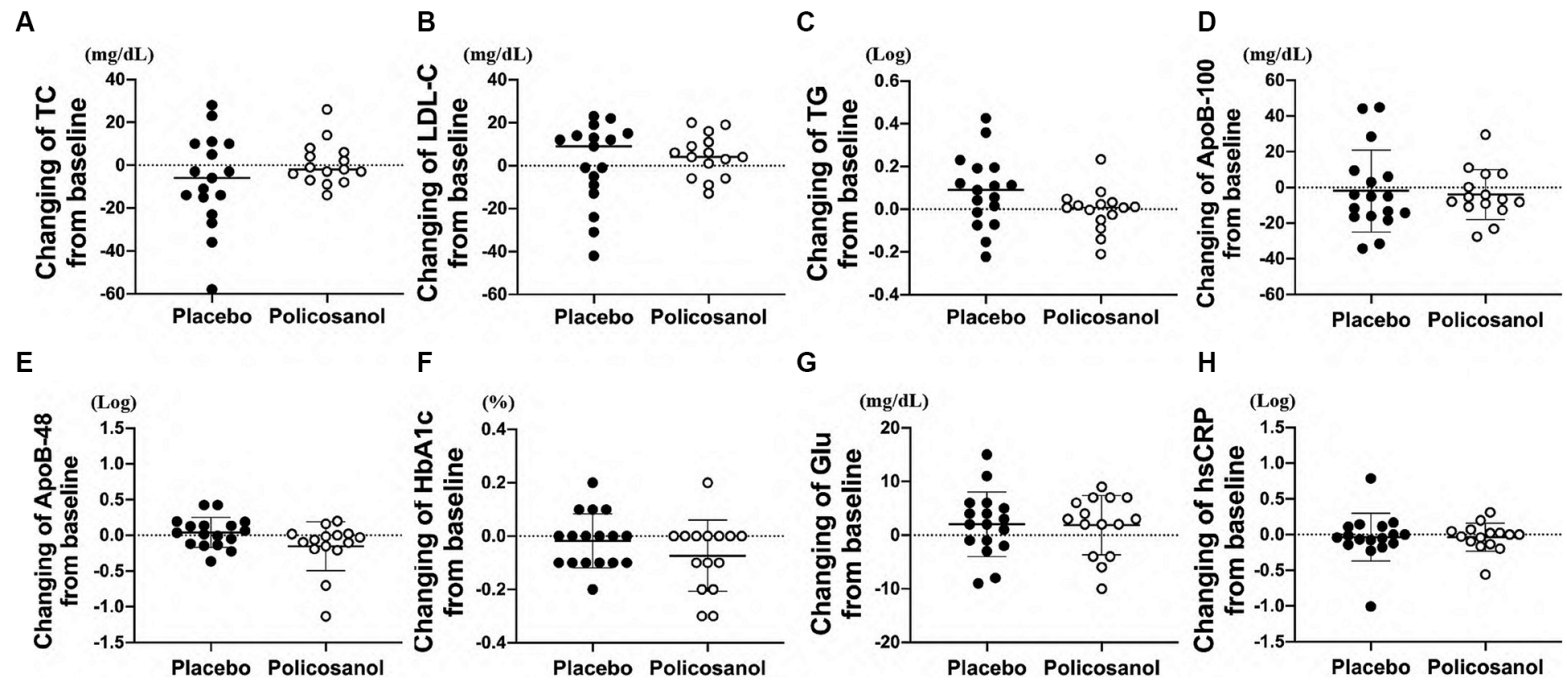
Effects of policosanol supplementation for 12 weeks on **(A)** TC, **(B)** LDL-C, **(C)** TG, **(D)** ApoB-100, **(E)** ApoB-48, **(F)** HbA1c, **(G)** Glu, and **(H)** hsCRP. The analysis was performed to evaluate the differences between the preintervention and postintervention values in the placebo and the policosanol group. Values represent the mean ± SD. TG and ApoB-48 and hsCRP were log-transformed prior to analysis. Placebo group, *n* = 17 and policosanol group, *n* = 15. TC, total cholesterol; LDL, low-density lipoprotein cholesterol; TG, triglyceride; Apo, apolipoprotein; HbA1c, hemoglobin A1c; Glu, fasting blood glucose, hsCRP, high sensitivity C-reactive protein.

Conversely, changes in the total cholesterol (Figure 2A), LDL-C (Figure 2B), and triglyceride (Figure 2C) levels from baseline were not different between the placebo and policosanol groups at week 12. No difference in the ApoB-100 (Figure 2D) and ApoB-48 (Figure 2E) levels, located on the surface of VLDL/LDL and chylomicron, respectively, was also found between the placebo and policosanol groups. At week 12, glucose-related factor levels such as HbA1c (Figure 2F) and blood glucose (Figure 2G) and the inflammatory factor hsCRP (Figure 2H) did not differ between the two groups.

### 3.2 Policosanol altered HDL morphology/lipid content

**Figure 3:**
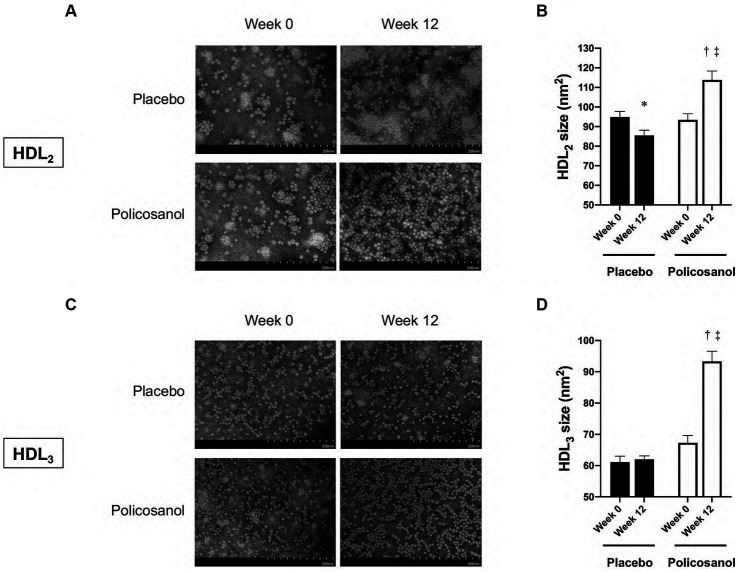
The transmission electron microscopy images and area analysis of HDL2 and HDL3 from the placebo and policosanol groups **(A,C)** and size comparison between the groups **(B,D)** between weeks 0 and 12. Values are presented as mean ± standard error of mean. ^*^p < 0.05 vs. placebo week 0, ^†^*p* < 0.001; placebo week 12, ^‡^*p* < 0.001 vs. policosanol week 0. Placebo group, *n* = 15 and policosanol group, *n* = 15. HDL, high-density lipoprotein.

Next, HDL form and composition alterations induced by policosanol were evaluated as policosanol particularly affected HDL. After lipoprotein isolation using the ultracentrifugation method, transmission electron microscopy revealed that the HDL particle number in the policosanol group at week 12 was higher, with more distinct particle morphology than that at week 0 (Figure 3A). Contrarily, the placebo group failed to show any notable change in particle morphology between weeks 0 and 12. In the policosanol group, the HDL_2_ particle size at week 12 was 22% higher than that of week 0 (*p* < 0.001). The placebo group at week 12 revealed a 10% decrease in particle size at week 12 than at week 0 (*p* = 0.020) with a more aggregated pattern than that of the policosanol group at week 12 (Figures 3A,B). No remarkable difference in the cholesterol and triglyceride levels in the HDL_2_ composition was noted in the policosanol in Table 2. Additionally, fluorescence intensity (FI) indicated glycation extent of yellowish fluorescence. Because the lower FI of HDL in the policosanol group was shown, it indicated that policosanol modulated a decreased extent of glycation in HDL.

**Table 2:** Analysis of lipid composition of the HDL changes from week 0 to week 12 in the placebo and policosanol groups.

		Placebo (*n* = 15)		Policosanol (*n* = 15)	
		Week 0	Week 12	Week 0	Week 12
	Cholesterol, mg/dL (in 2 mg/mL of protein)	82.3 ± 0.8	79.0 ± 1.2^†^	85.7 ± 3.6	82.5 ± 2.8
HDL_2_	Triglyceride, mg/dL (in 2 mg/mL of protein)	11.9 ± 0.3	13.8 ± 0.6^*^	14.4 ± 0.5	12.9 ± 0.6
	FI, Glycated	2,452 ± 91	2,453 ± 171	2,568 ± 225	2,152 ± 204^*^
	Cholesterol, mg/dL (in 2 mg/mL of protein)	59.8 ± 1.9	41.3 ± 4.1^*^	59.7 ± 2.7	61.3 ± 1.8
HDL_3_	Triglyceride, mg/dL (in 2 mg/mL of protein)	6.7 ± 0.6	6.2 ± 0.8	9.4 ± 0.5	7.1 ± 0.4^*^
	FI, Glycated	1817 ± 149	1911 ± 309	2,115 ± 197	1811 ± 154^*^

Values represent the mean ± standard error of the mean. ^*^p < 0.05; ^†^*p* < 0.01 vs. week 0 in each group. Placebo group, *n* = 15 and policosanol group, *n* = 15. HDL, high-density lipoprotein; FI, fluorescence intensity (Ex = 370 nm, Em = 440 nm, 0.01 mg/mL of protein).

In Figure 3C, transmission electron microscopy revealed that at week 12, the HDL_3_ particle size in the policosanol group was 1.4-fold higher than that at week 0 (*p* < 0.001), with a more distinct particle shape. The placebo group failed to show a notable change in HDL_3_ particle morphology. In Table 2, the policosanol group showed almost no change, even a slight increase in the cholesterol content in HDL_3_ between weeks 0 and 12, whereas the placebo group revealed a 31% decrease in HDL_3_ between weeks 0 and 12. Interestingly, at week 12, the triglyceride content in the policosanol group was 25% lower than that at week 0, whereas the placebo group demonstrated no notable change between weeks 0 and 12. These results indicate that policosanol supplementation also affects HDL_3_ size enlargement, whereas the placebo group revealed no difference in particle size.

Furthermore, to verify the cholesterol and triglyceride contents in lipoproteins defined by particle size in detail, HPLC analysis was performed (28, 29). At baseline, 20 lipoprotein fractions from a chylomicron to HDL are shown in Table 3. The difference at baseline was only observed in the cholesterol content of VLDL (fraction 5) and triglyceride content of HDL (fraction 17). However, it was deemed to be unimportant because the values were of very low proportion compared to the total amount in both cholesterol and triglyceride.

**Table 3:** Baseline characteristics of the lipoprotein subfraction analyzed by HPLC.

	Placebo (*n* = 17)		Policosanol (*n* = 15)	
	Cholesterol	Triglyceride	Cholesterol	Triglyceride
Total, mg/dL	211.0 ± 21.0	81.2 [58.7–96.9]	207.5 ± 15.5	92.9 [67.0–127.2]
Large CM (Fraction 1), mg/dL	0.09 [0.06–0.16]	0.28 [0.13–0.72]	0.13 [0.07–0.23]	0.28 [0.18–1.22]
CM (Fraction 2), mg/dL	0.07 [0.03–0.12]	0.45 [0.17–1.02]	0.08 [0.04–0.17]	0.54 [0.4–1.79]
Largest VLDL (Fraction 3), mg/dL	0.45 [0.33–0.62]	2.8 [1.3–5.0]	0.59 [0.41–1.12]	3.7 [2.3–9.4]
Very Large VLDL (Fraction 4), mg/dL	1.05 [0.52–1.51]	9.8 [4.5–13.0]	1.22 [0.72–3.18]	11.9 [6.8–21.5]
Large VLDL (Fraction 5), mg/dL	5.8 [5.1–6.7]	16.5 [9.2–23.1]	7.5 [5.8–8.5]^*^	22.6 [12.0–29.2]
Medium VLDL (Fraction 6), mg/dL	5.3 [4.3–9.0]	12.7 [8.3–17.1]	7.3 [5.0–8.7]	17.0 [12.1–20.0]
Small VLDL (Fraction 7), mg/dL	8.2 [6.3–10.9]	5.5 [4.7–6.0]	8.1 [7.1–10.6]	6.7 [5.1–7.4]
Large LDL (Fraction 8), mg/dL	35.6 ± 5.8	8.1 [7.3–10.1]	33.8 ± 5.9	9.1 [7.6–10.3]
Medium LDL (Fraction 9), mg/dL	55.2 ± 5.5	7.8 [7.2–9.1]	53.3 ± 4.6	8.5 [7.6–9.4]
Medium to small LDL (Fraction 10), mg/dL	24.5 ± 3.6	3.3 [3.0–3.8]	23.6 ± 3.3	3.4 [2.7–4.1]
Small LDL (Fraction 11), mg/dL	7.8 ± 1.1	1.00 [0.91–1.21]	7.4 ± 0.9	1.03 [0.87–1.26]
Very small LDL (Fraction 12), mg/dL	1.3 ± 0.3	0.23 [0.18–1.28]	1.2 ± 0.2	0.21 [0.19–0.3]
Smallest LDL (Fraction 13), mg/dL	1.2 ± 0.2	0.19 [0.17–0.23]	1.2 ± 0.1	0.22 [0.17–0.26]
Largest HDL (Fraction 14), mg/dL	0.9 ± 0.2	0.16 [0.14–0.19]	0.9 ± 0.2	0.17 [0.15–0.23]
Very Large HDL (Fraction 15), mg/dL	3.0 ± 1.5	0.50 [0.37–0.76]	3.1 ± 1.4	0.69 [0.57–0.9]
Large HDL (Fraction 16), mg/dL	15.1 ± 6.1	2.7 [1.9–3.5]	14.9 ± 6.3	3.3 [2.7–4.1]
Medium HDL (Fraction 17), mg/dL	22.0 ± 3.4	3.1 [2.7–3.7]	21.0 ± 3.8	4.0 [3.1–5.8]^*^
Small HDL (Fraction 18), mg/dL	14.9 ± 1.8	1.9 [1.5–2.1]	14.6 ± 1.8	2.5 [1.8–3.2]
Very Small HDL (Fraction 19), mg/dL	4.8 ± 0.5	0.47 [0.42–0.6]	4.6 ± 0.6	0.64 [0.46–0.94]
Smallest HDL (Fraction 20), mg/dL	1.5 ± 0.1	0.56 [0.52–0.64]	1.5 ± 0.1	0.60 [0.56–0.69]

Values represent the mean ± standard deviation or median with interquartile range. Values of fractions 1–7 of cholesterol and all fractions of triglyceride in both groups were log-transformed prior to analysis. ^*^p < 0.05 vs. cholesterol or triglyceride values in the opposite group. Placebo group, *n* = 17 and policosanol group, *n* = 15. CM, chylomicron; VLDL, very low-density lipoprotein cholesterol; LDL, low-density lipoprotein cholesterol; HDL, high-density lipoprotein.

**Figure 4:**
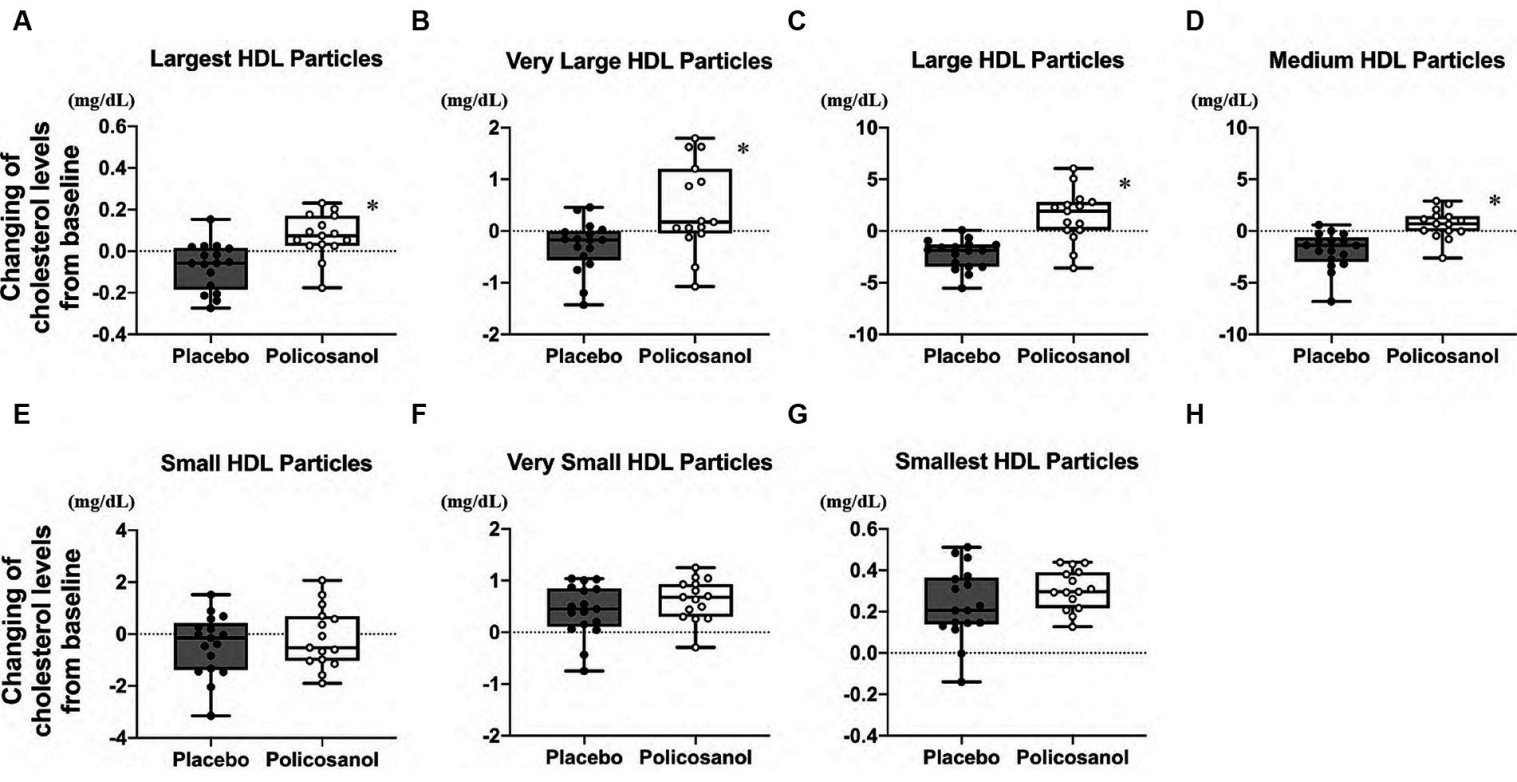
Cholesterol level analysis through HDL particle size using the high-performance liquid chromatography (HPLC) method after policosanol supplementation for 12 weeks. The data showed the **(A)** largest HDL particles, **(B)** very large HDL particles, **(C)** large HDL particles, **(D)** medium HDL particles, **(E)** small HDL particles, **(F)** very small HDL particles, and **(G)** smallest HDL particles. The analysis was performed to evaluate the differences between the preintervention and postintervention values in the placebo and the policosanol group. Values represent the mean and minimum to maximum. ^*^p < 0.01 vs. placebo. Placebo group, *n* = 17 and policosanol group, *n* = 15. HDL, high-density lipoprotein.

**Figure 5:**
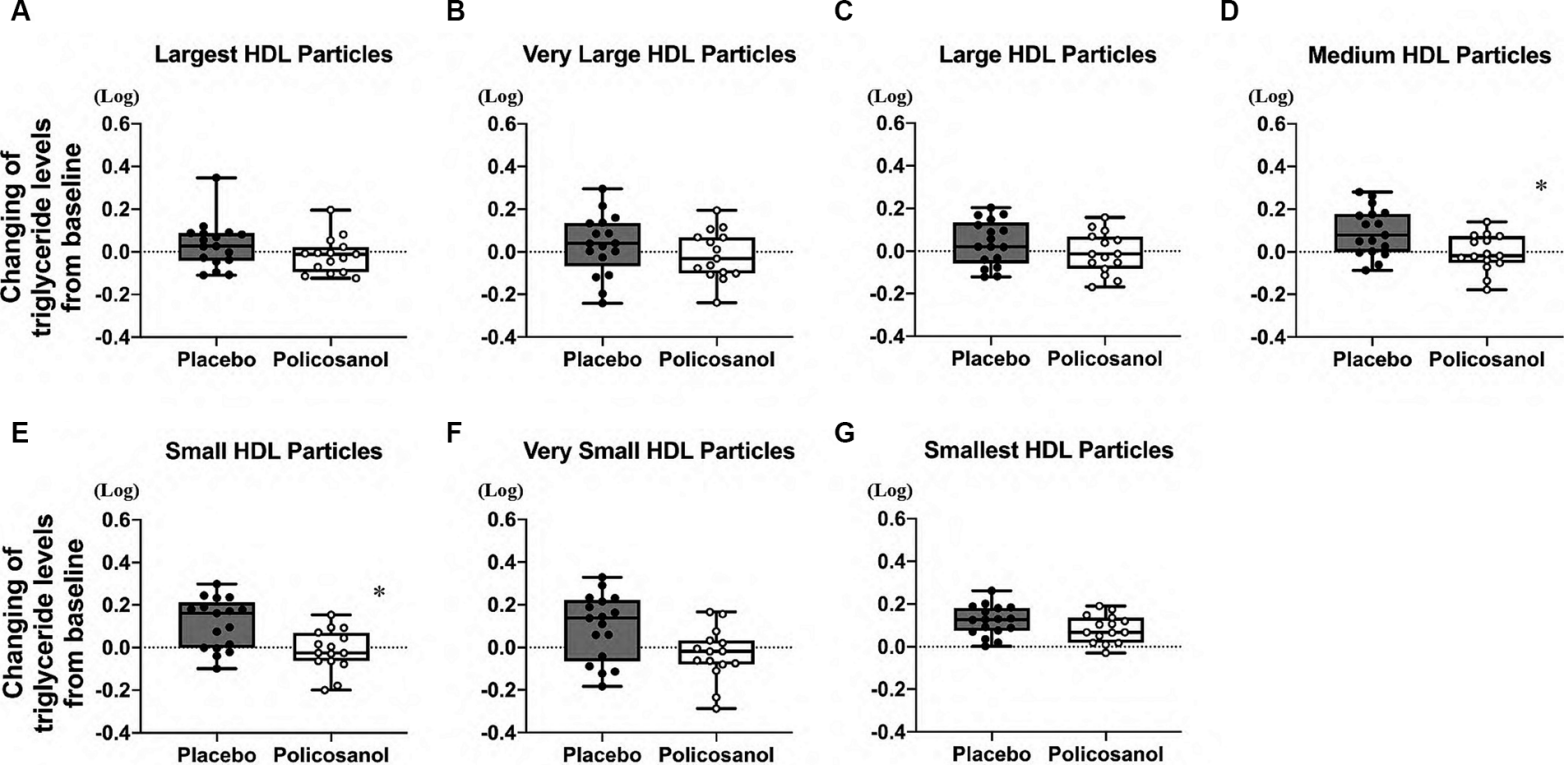
Triglyceride level analysis by HDL particle size using the HPLC method after policosanol supplementation for 12 weeks. The data reveal the **(A)** largest HDL, **(B)** very large HDL, **(C)** large HDL, **(D)** medium HDL, **(E)** small HDL, **(F)** very small HDL, and **(G)** smallest HDL particles. The analysis was conducted to assess the differences between the preintervention and postintervention values in the placebo and the policosanol group. Values represent the mean and minimum to maximum. In both groups, triglyceride values of all fractions were log-transformed prior to analysis. ^*^p < 0.05 vs. placebo. Placebo group, *n* = 17 and policosanol group, *n* = 15. HDL, high-density lipoprotein.

The alterations in cholesterol content in each HDL subclass (largest to smallest HDL particles with seven fractions) were shown in Figure 4. The HPLC analysis revealed that policosanol increased the cholesterol content in the largest to medium HDL particles (four fractions) (Figures 4A–D), except in small HDL particles (Figures 4E–G), compared with the placebo. As shown in Figure 5, triglyceride content changes from baseline was reduced in the policosanol group in terms of the medium to small HDL particles (17–18 fractions); however, no change was noted in other HDL fractions. These microscopic and HPLC outcomes revealed that policosanol induced the growth of larger HDL particles, increased the cholesterol content of larger HDL particles, and reduced the triglyceride content of smaller HDL particles.

In terms of the changes in other lipoproteins induced by policosanol supplementation, cholesterol and triglyceride in the chylomicron (fractions 1–2), VLDL (fractions 3–7), and LDL (fractions 8–13) were slightly different at week 12 (Supplementary Figure S1). Cholesterol content in the medium-sized VLDL (fraction 6, Supplementary Figure S1F) and smallest LDL (fraction 13, Supplementary Figure S1M) was decreased and increased, respectively. However, the difference was believed to be nonsignificant because (1) there were no remarkable alterations in the total LDL-C levels (Figure 2B), (2) the cholesterol levels in two fractions were lower compared with the total cholesterol levels, and (3) same difference was not observed in the consecutive fractions. As shown in Supplementary Figure S2, the triglyceride content in large to medium to small VLDL (fractions 5–7) was decreased (Supplementary Figures S2E–G) in the policosanol group, as compared with the placebo.

### 3.3 Policosanol has effects on HDL function, especially in the high HDL-C group

We further examined whether HDL-C levels at baseline were associated with policosanol effects, such as the changes in the HDL CEC and cholesterol/component proteins in HDL. In Table 4, data of four groups stratified by baseline (week 0) HDL-C levels (placebo with low HDL-C levels, *n* = 8; placebo with high HDL-C levels, *n* = 9; policosanol with low HDL-C levels, *n* = 8; policosanol with high HDL-C levels, *n* = 7) are presented.

**Table 4:** Baseline characteristics of the four groups categorized as low or high HDL-C levels at week 0 in the placebo and policosanol groups.

	Placebo		Policosanol	
	Low (*n* = 8)	High (*n* = 9)	Low (*n* = 8)	High (*n* = 7)
HDL-cholesterol, mg/dL	57.6 ± 5.5	73.7 ± 5.9^† §^	56.0 ± 6.3	72.6 ± 9.3^* §^
HDL efflux capacity, %	15.0 ± 0.6	15.9 ± 0.5	15.3 ± 0.5	16.7 ± 1.0^† ‡^
Total cholesterol, mg/dL	210.3 ± 15.2	218.9 ± 16.3	213.3 ± 16.4	224.4 ± 9.8
LDL-cholesterol, mg/dL	137.1 ± 8.5	131.6 ± 12.9	136.8 ± 13.5	124.1 ± 10.4
Triglycerides, mg/dL^a^	118.0 [69.8–136.0]	58.0 [53.0–82.0]	122.5 [81.3–188.8]	50.0 [50.0–109.0]
Apolipoprotein AI, mg/dL	160.0 ± 17.6	178.9 ± 13.2	169.3 ± 16.7	196.3 ± 18.0^† ‡^
Apolipoprotein AII, mg/dL	32.7 ± 4.3	36.0 ± 4.4	34.8 ± 3.4	34.1 ± 2.7
Apolipoprotein B100, mg/dL	78.3 ± 19.8	79.0 ± 29.0	86.0 ± 18.6	81.7 ± 12.2
Apolipoprotein B48, mg/dL^a^	0.28 [0.22–1.02]	0.43 [0.26–0.9]	0.26 [0.17–0.79]	0.35 [0.19–0.55]

Values represent the mean ± standard deviation or median with interquartile range.

a Triglyceride and apolipoprotein B-48 in both groups were log-transformed prior to analysis. ^*^p < 0.05; ^†^*p* < 0.01 vs. placebo low HDL-C group, ^‡^*p* < 0.05; ^§^*p* < 0.01 vs. policosanol low HDL-C group.

**Figure 6:**
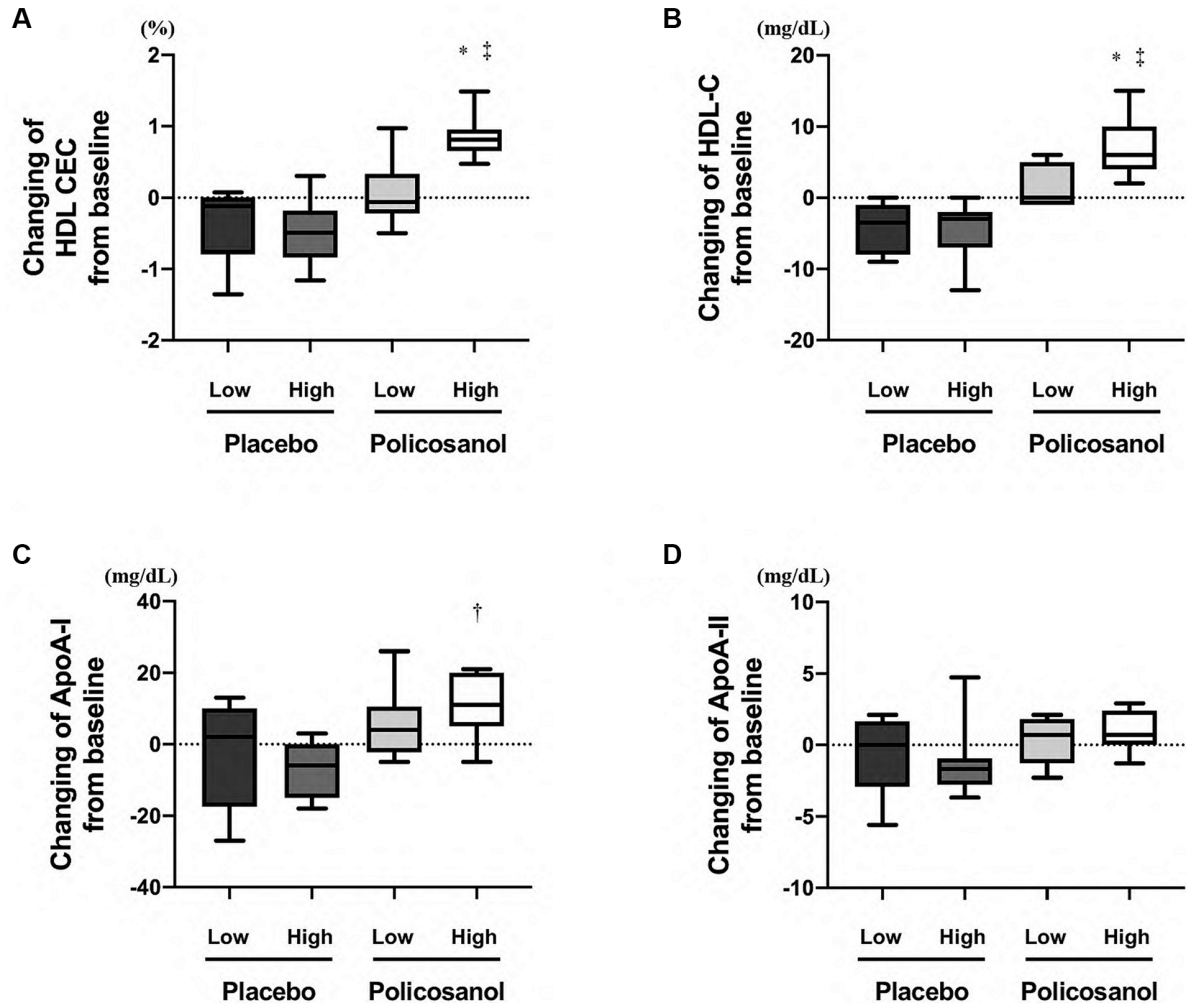
The expression of policosanol effects in the four groups stratified by baseline HDL-C levels, targeting for **(A)** HDL CEC and **(B)** HDL-C, **(C)** ApoA-I, and **(D)** ApoA-II levels. The analysis was performed to assess the differences between the preintervention and postintervention values in the placebo and the policosanol group. Values represent the mean and minimum to maximum. ^*^p < 0.01 vs. low HDL-C levels at baseline in the placebo group, ^†^*p* < 0.05; ^‡^*p* < 0.01 vs. high HDL-C levels at baseline in the placebo group. HDL, high-density lipoprotein; CEC, cholesterol efflux capacity; Apo, apolipoprotein; Low, low HDL-C levels at baseline; High, high HDL-C levels at baseline.

Comparing the four groups at week 12, HDL CEC was upregulated in only the high-HDL-C group with policosanol, as compared with the other three groups (Figure 6A). Likewise, HDL-C (Figure 6B) and ApoA-I (Figure 6C) levels were elevated in only the high HDL-C group of policosanol supplementation. However, policosanol did not change the ApoA-II levels in the four groups stratified by HDL-C levels at week 0 (Figure 6D).

Similarly, the sex-specific effects of policosanol supplementation were explored in four groups, as shown in Supplementary Table S1 (males in the placebo group, *n* = 9; females in the placebo group, *n* = 8; males in the policosanol group, *n* = 8, females in the policosanol group, *n* = 7). No difference between the four groups was noted at baseline. Surprisingly, policosanol supplementation increased the HDL CEC and ApoA-I levels in only females with policosanol and HDL-C levels in both sexes with policosanol compared to that in placebo (Supplementary Figures S3A–C). However, ApoA-II levels were not altered in four groups (Supplementary Figure S3D).

### 3.4 Changes in HDL CEC were more strongly associated with HDL-C and ApoA-I changes than with changes in ApoA-II in healthy participants

**Figure 7:**
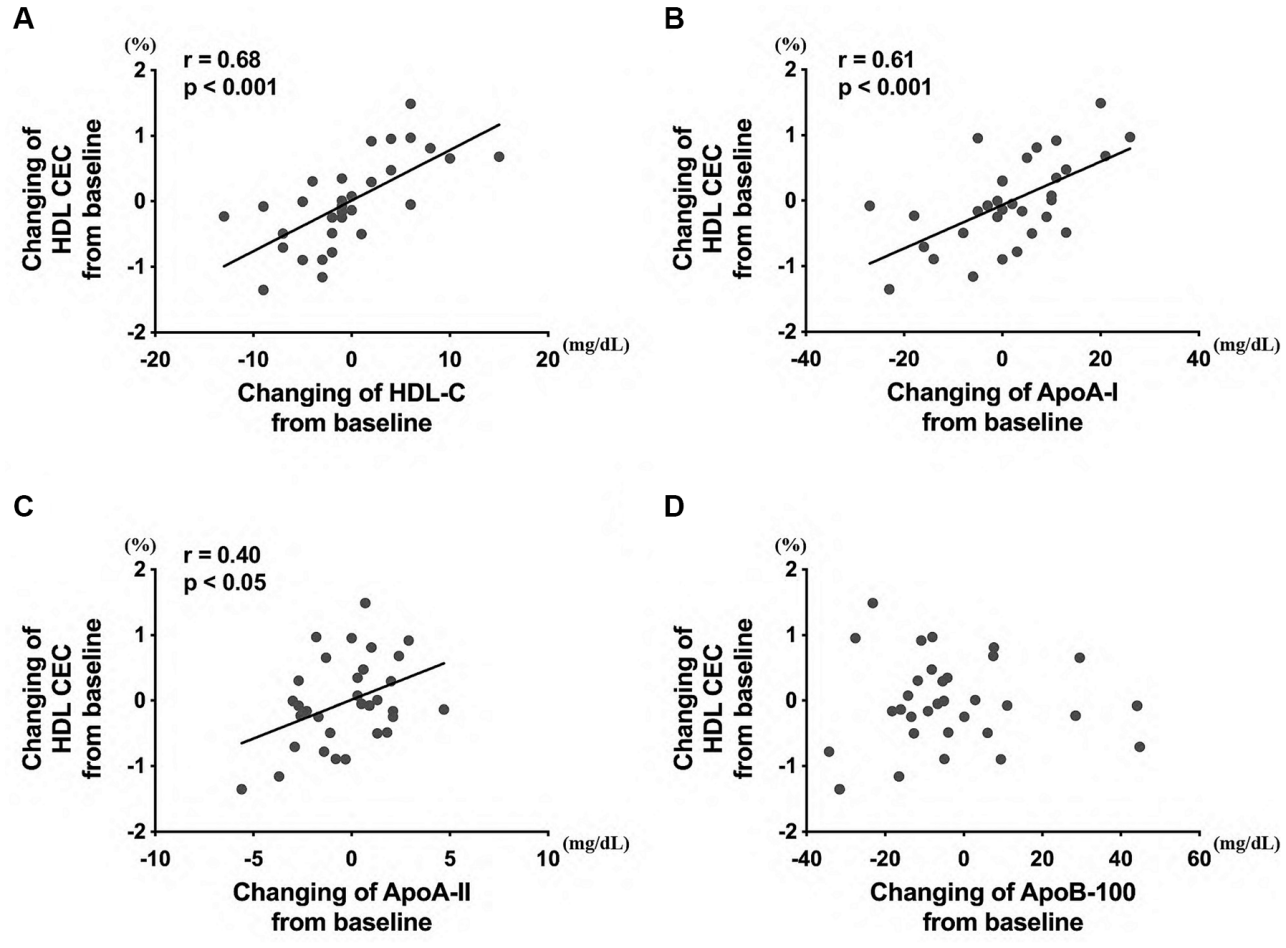
Correlation between the HDL CEC and HDL/LDL-related factors, including **(A)** HDL-C, **(B)** ApoA-I, **(C)** ApoA-II, and **(D)** ApoB-100 levels. The analysis was performed to evaluate the differences between the preintervention and postintervention values in the placebo and the policosanol group. HDL, high-density lipoprotein; CEC, cholesterol efflux capacity; LDL, low-density lipoprotein cholesterol; Apo, apolipoprotein.

Finally, to determine whether the values were related to the alteration of other HDL/LDL-related factors in all healthy participants, changes in HDL CEC from week 0 to week 12 were examined. The HDL CEC alteration was associated with changes in HDL-C (Figure 7A), ApoA-I (Figure 7B), and ApoA-II (Figure 7C) levels, but not in ApoB-100 levels (Figure 7D).

## 4 Discussion

This study showed that policosanol supplementation (20 mg/day, 12 weeks) induced the elevation of HDL-C levels and HDL CEC, together with larger-size HDL and its cholesterol content in Japanese healthy participants. Particularly, individuals with high HDL-C concentrations at baseline (>64 mg/dL in this study) demonstrated a tendency of upregulation of their HDL capacity via policosanol. HDL CEC fluctuation is especially associated with HDL-C and ApoA-I changes in the normal ranges. Thus, HDL-C and ApoA-I may be useful in identifying the HDL functional changes through policosanol supplementation.

HDL CEC is a more important indicator for ASCVD than HDL-C levels globally (30, 35) and in Japan (36). HDL CEC is also known to be correlated with HDL remodeling and inflammation (37). In human studies, under such circumstances, food, drinks, and other supplementations such as a Mediterranean diet (enriched with virgin olive oil or enriched with nuts) (38), olive oil polyphenols (39), coffee (40), and anthocyanin (41) are reported to increase HDL CEC. In the same manner, this study also revealed that HDL CEC is increased by policosanol supplementation in Japanese healthy participants (Figure 1A). From prior reports citing that policosanol increased blood HDL-C levels in human studies (22) and recombinant HDL containing policosanol upregulated cholesterol efflux in an *in vitro* study (42), policosanol was anticipated to easily interact with HDL, and this hypothesis corroborates with this study’s results. Additionally, participants with high HDL-C levels at baseline received the benefits of policosanol, as compared to those with low HDL-C levels at baseline (Figure 6). Furthermore, policosanol supplementation affected HDL CEC and ApoA-I levels especially in females, despite an increase in HDL-C levels in both sexes (Supplementary Figure S3). Because the included participants are generally healthy and had normal HDL-C levels of >40 mg/dL, the outcome for the non-healthy participants with low or very high HDL-C levels may differ. The HDL CEC is known to be correlated with HDL-C and ApoA-I concentrations in healthy participants (30). Whereas, several data showed a negative correlation between HDL CEC and HDL-C/ApoA-1 concentration, especially in cases with genetic mutations (43), weight changes (44), and intake of the drug probucol (45). This study revealed the positive relationship between HDL CEC and HDL-C/ApoA-I concentration (Figure 7), which is consistent with previous research (30). Moreover, HDL-C changes by policosanol supplementation may alter HDL CEC because HDL CEC fluctuation was highly correlated with HDL-C level alterations in the present study (Figure 7).

HDL concentration reduction is partially known to be related to aging (46); however, some centenarians presented with higher HDL-C levels (47), suggesting the association between high HDL levels, anti-atherosclerosis, and longevity. Policosanol supplementation reportedly elevated the HDL-C levels remarkably (48) and tendentially (49) in animal models and significantly in human studies (15, 22, 50). In this study, the effect of policosanol in increasing the HDL-C levels concurs with the above reports (Figure 1B). In Japanese, the trend of an increase in HDL-C concentration has been reported to be high worldwide (51). In the present study, the mean HDL-C levels of healthy participants at baseline were approximately 65 mg/dL (Table 1), which were similar with the mean Japanese HDL-C concentration in recent years (52); thus, these policosanol effects are believed to reflect the real-world setting in Japan because HDL-C levels of the participants matched recent data. Although extremely high HDL-C levels are recently suggested to have a risk for mortality (53), policosanol does not have a sufficient power to extremely increase the HDL-C levels. Thus, policosanol supplementation may elevate HDL-C levels along with a higher HDL CEC.

There are many factors related to HDL-C concentration, which include cholesteryl ester transfer protein (CETP), lecithin–cholesterol acyl transferase, endothelial lipase, inflammation, and a gene mutation background (54, 55). Thus, understanding the health condition fully from HDL-C levels alone after the above factors were found to be closely linked to each other becomes harder. CETP, one of the HDL remodeling enzymes, plays a role in the exchange between cholesteryl ester of HDL and triglyceride of apolipoprotein B100-containing lipoproteins (such as VLDL and LDL) (56). Along with the increase in the HDL CEC, use of olive oil polyphenols led to larger HDL2 particles (39). Moreover, a Mediterranean diet enriched with virgin olive oil reduces CETP activity with higher HDL CEC in a human study (38), whereas the current study revealed that policosanol supplementation has larger and cholesterol-rich HDL particles (Figures 3, 4) with elevated HDL CEC (Figure 1). Some reports showed that policosanol reduced the CETP activity in animal (48) and human studies (42, 57). Generally, the decline in the CETP activity by CETP inhibitors induces high cholesterol levels of HDL (HDL-C) and low triglyceride levels of VLDL (58) and HDL (59), which are consistent with the present outcomes (Figures 5D,E; Supplementary Figures S2E–G). Moreover, one of the CETP inhibitors demonstrated increases not only in the HDL-C levels but also in the HDL CEC (60). Extensively, CETP may be highly related to policosanol effects in HDL morphology and functional changes. Additionally, CETP and cholesterol esters are present in large amounts in larger HDL (61). These cholesterol esters are reported to be derived from the liver during large HDL particle synthesis from the human tracer study (61), and policosanol may be involved in HDL synthesis in the liver.

In terms of LDL, an *in vitro* research previously suggested that policosanol inhibits hepatic cholesterol biosynthesis during the first step of the cholesterol generation pathway (62). Additionally, some studies reported that policosanol reduces LDL-C levels. However, in the current study, policosanol supplementation failed to reduce the LDL-C levels (Figure 2B; Supplementary Figures S1H–M). The rationale for the absence of any effects on LDL-C may be attributed to the property of policosanol in healthy participants, which can especially affect HDL more as compared to LDL.

This study has several limitations. First, the number of the included participants was limited. Second, the association between policosanol effects and HDL-related factors, except for ApoA-I and ApoA-II (e.g., CETP and other factors that modulate HDL) remained unclear as measurements were not performed. Third, clinical trials were conducted using Cuban policosanol; however, whether other policosanols would also show similar results, such as elevations of HDL CEC, remained unclear. Fourth, the background of policosanol metabolism, including uptake and turnover in the human body, remains poorly elucidated.

Further investigations are warranted to establish the evidence of policosanol’s effects in the clinical setting.

## Data availability statement

The original contributions presented in the study are included in the article/Supplementary material, further inquiries can be directed to the corresponding author.

## Ethics statement

The studies involving humans were approved by the ethics committee of the Koseikai Fukuda Internal Medicine Clinic (Osaka, Japan, IRB approval number 15000074, approval date on September 18, 2021). The studies were conducted in accordance with the local legislation and institutional requirements. The participants provided their written informed consent to participate in this study.

## Author contributions

YU: Conceptualization, Formal analysis, Funding acquisition, Investigation, Project administration, Resources, Supervision, Writing – review & editing. TK: Writing – original draft. KS: Investigation, Writing – review & editing. SA: Investigation, Writing – review & editing. SN: Investigation, Writing – review & editing. TY: Investigation, Writing – review & editing. J-EK: Investigation, Writing – review & editing. K-HC: Writing – review & editing.

## Footnotes

^1^ http://rsb.info.nih.gov/ij/

## References

[1] Ko DT Alter DA Guo H Koh M Lau G Austin PC. High-density lipoprotein cholesterol and cause-specific mortality in individuals without previous cardiovascular conditions: the CANHEART study. J Am Coll Cardiol. (2016) 68:2073–83. doi: 10.1016/j.jacc.2016.08.038, PMID: 27810046

[2] Emerging Risk Factors Collaboration Di Angelantonio E Sarwar N Perry P Kaptoge S Ray KK. Major lipids, apolipoproteins, and risk of vascular disease. JAMA. (2009) 302:1993–2000. doi: 10.1001/jama.2009.1619, PMID: 19903920 PMC3284229

[3] Kannel WB. High-density lipoproteins: epidemiologic profile and risks of coronary artery disease. Am J Cardiol. (1983) 52:B9–B12. doi: 10.1016/0002-9149(83)90649-5 6577783

[4] Matsuzaki M Kita T Mabuchi H Matsuzawa Y Nakaya N Oikawa S. Large scale cohort study of the relationship between serum cholesterol concentration and coronary events with low-dose simvastatin therapy in Japanese patients with hypercholesterolemia. Circ J. (2002) 66:1087–95. doi: 10.1253/circj.66.1087 12499611

[5] Haase CL Tybjaerg-Hansen A Qayyum AA Schou J Nordestgaard BG Frikke-Schmidt R. LCAT, HDL cholesterol and ischemic cardiovascular disease: a Mendelian randomization study of HDL cholesterol in 54,500 individuals. J Clin Endocrinol Metab. (2012) 97:E248–56. doi: 10.1210/jc.2011-1846 22090275

[6] Voight BF Peloso GM Orho-Melander M Frikke-Schmidt R Barbalic M Jensen MK. Plasma HDL cholesterol and risk of myocardial infarction: a mendelian randomisation study. Lancet. (2012) 380:572–80. doi: 10.1016/S0140-6736(12)60312-2, PMID: 22607825 PMC3419820

[7] Soria-Florido MT Schroder H Grau M Fito M Lassale C. High density lipoprotein functionality and cardiovascular events and mortality: A systematic review and meta-analysis. Atherosclerosis. (2020) 302:36–42. doi: 10.1016/j.atherosclerosis.2020.04.015, PMID: 32438197

[8] Phillips MC. Molecular mechanisms of cellular cholesterol efflux. J Biol Chem. (2014) 289:24020–9. doi: 10.1074/jbc.R114.583658 25074931 PMC4148835

[9] Lewis GF Rader DJ. New insights into the regulation of HDL metabolism and reverse cholesterol transport. Circ Res. (2005) 96:1221–32. doi: 10.1161/01.RES.0000170946.56981.5c, PMID: 15976321

[10] Attie AD Kastelein JP Hayden MR. Pivotal role of ABCA1 in reverse cholesterol transport influencing HDL levels and susceptibility to atherosclerosis. J Lipid Res. (2001) 42:1717–26. doi: 10.1016/S0022-2275(20)31498-X, PMID: 11714841

[11] Ebtehaj S Gruppen EG Bakker SJL Dullaart RPF Tietge UJF. HDL (high-density lipoprotein) cholesterol efflux capacity is associated with incident cardiovascular disease in the general population. Arterioscler Thromb Vasc Biol. (2019) 39:1874–83. doi: 10.1161/ATVBAHA.119.312645 31315436

[12] Hisauchi I Ishikawa T Ayaori M Uto-Kondo H Koshikawa Y Ukaji T. High-density lipoprotein cholesterol efflux capacity as a novel prognostic surrogate for coronary artery disease. J Atheroscler Thromb. (2021) 28:696–702. doi: 10.5551/jat.59279, PMID: 32908115 PMC8265426

[13] Qiu C Zhao X Zhou Q Zhang Z. High-density lipoprotein cholesterol efflux capacity is inversely associated with cardiovascular risk: a systematic review and meta-analysis. Lipids Health Dis. (2017) 16:212. doi: 10.1186/s12944-017-0604-5 29126414 PMC5681808

[14] Chow SL Bozkurt B Baker WL Bleske BE Breathett K Fonarow GC. Complementary and alternative medicines in the management of heart failure: a scientific statement from the American Heart Association. Circulation. (2023) 147:e4–e30. doi: 10.1161/CIR.0000000000001110, PMID: 36475715

[15] Cho KH Nam HS Baek SH Kang DJ Na H Komatsu T. Beneficial effect of cuban policosanol on blood pressure and serum lipoproteins accompanied with lowered glycated hemoglobin and enhanced high-density lipoprotein functionalities in a randomized, placebo-controlled, and double-blinded trial with healthy japanese. Int J Mol Sci. (2023) 24:5185. doi: 10.3390/ijms24065185, PMID: 36982259 PMC10048825

[16] Park HJ Yadav D Jeong DJ Kim SJ Bae MA Kim JR. Short-term consumption of cuban policosanol lowers aortic and peripheral blood pressure and ameliorates serum lipid parameters in healthy korean participants: randomized, double-blinded, and placebo-controlled study. Int J Environ Res Public Health. (2019) 16:809. doi: 10.3390/ijerph16050809, PMID: 30841655 PMC6427682

[17] Kabir Y Kimura S. Biodistribution and metabolism of orally administered octacosanol in rats. Ann Nutr Metab. (1993) 37:33–8. doi: 10.1159/000177746, PMID: 8470870

[18] Zhai Z Niu KM Liu H Lin C Tu Y Liu Y. Policosanol alleviates hepatic lipid accumulation by regulating bile acids metabolism in C57BL6/mice through AMPK-FXR-TGR5 cross-talk. J Food Sci. (2021) 86:5466–78. doi: 10.1111/1750-3841.15951 34730235

[19] Sharma R Matsuzaka T Kaushik MK Sugasawa T Ohno H Wang Y. Octacosanol and policosanol prevent high-fat diet-induced obesity and metabolic disorders by activating brown adipose tissue and improving liver metabolism. Sci Rep. (2019) 9:5169. doi: 10.1038/s41598-019-41631-1, PMID: 30914769 PMC6435753

[20] Zhai Z Liu J Niu KM Lin C Tu Y Liu Y. Integrated metagenomics and metabolomics to reveal the effects of policosanol on modulating the gut microbiota and lipid metabolism in hyperlipidemic C57BL/6 mice. Front Endocrinol (Lausanne). (2021) 12:722055. doi: 10.3389/fendo.2021.722055, PMID: 34707567 PMC8542985

[21] Askarpour M Ghaedi E Roshanravan N Hadi A Mohammadi H Symonds ME. Policosanol supplementation significantly improves blood pressure among adults: A systematic review and meta-analysis of randomized controlled trials. Complement Ther Med. (2019) 45:89–97. doi: 10.1016/j.ctim.2019.05.023, PMID: 31331588

[22] Gouni-Berthold I Berthold HK. Policosanol: clinical pharmacology and therapeutic significance of a new lipid-lowering agent. Am Heart J. (2002) 143:356–65. doi: 10.1067/mhj.2002.119997, PMID: 11835043

[23] Crespo N Illnait J Mas R Fernandez L Fernandez J Castano G. Comparative study of the efficacy and tolerability of policosanol and lovastatin in patients with hypercholesterolemia and noninsulin dependent diabetes mellitus. Int J Clin Pharmacol Res. (1999) 19:117–27. PMID: 10939029

[24] Castano G Fernandez L Mas R Illnait J Mesa M Fernandez JC. Comparison of the effects of policosanol and atorvastatin on lipid profile and platelet aggregation in patients with dyslipidaemia and type 2 diabetes mellitus. Clin Drug Investig. (2003) 23:639–50. doi: 10.2165/00044011-200323100-00003, PMID: 17535079

[25] Kinoshita M Yokote K Arai H Iida M Ishigaki Y Ishibashi S. Japan Atherosclerosis Society (JAS) guidelines for prevention of atherosclerotic cardiovascular diseases 2017. J Atheroscler Thromb. (2018) 25:846–984. doi: 10.5551/jat.GL2017, PMID: 30135334 PMC6143773

[26] Havel RJ Eder HA Bragdon JH. The distribution and chemical composition of ultracentrifugally separated lipoproteins in human serum. J Clin Invest. (1955) 34:1345–53. doi: 10.1172/JCI103182, PMID: 13252080 PMC438705

[27] Cho KH Kim JR Lee IC Kwon HJ. Native high-density lipoproteins (HDL) with higher paraoxonase exerts a potent antiviral effect against SARS-CoV-2 (COVID-19), while glycated HDL lost the antiviral activity. Antioxidants (Basel). (2021) 10:209. doi: 10.3390/antiox10020209 33535459 PMC7912765

[28] Okazaki M Yamashita S. Recent advances in analytical methods on lipoprotein subclasses: calculation of particle numbers from lipid levels by gel permeation HPLC Using “Spherical Particle Model”. J Oleo Sci. (2016) 65:265–82. doi: 10.5650/jos.ess16020, PMID: 27041512

[29] Yokoyama S Remaley AT Sampson M Ai M Okazaki M. Validation by HPLC analyses of new equations for estimating cholesterol in plasma lipoprotein subfractions. Biochim Biophys Acta Mol Cell Biol Lipids. (2021) 1866:158986. doi: 10.1016/j.bbalip.2021.158986, PMID: 34102316

[30] Khera AV Cuchel M de la Llera-Moya M Rodrigues A Burke MF Jafri K. Cholesterol efflux capacity, high-density lipoprotein function, and atherosclerosis. N Engl J Med. (2011) 364:127–35. doi: 10.1056/NEJMoa1001689, PMID: 21226578 PMC3030449

[31] Komatsu T Abe S Nakashima S Sasaki K Higaki Y Saku K. Dipeptidyl peptidase-4 inhibitor sitagliptin phosphate accelerates cellular cholesterol efflux in THP-1 cells. Biomolecules. (2023) 13:228. doi: 10.3390/biom13020228, PMID: 36830597 PMC9953524

[32] Uehara Y Engel T Li Z Goepfert C Rust S Zhou X. Polyunsaturated fatty acids and acetoacetate downregulate the expression of the ATP-binding cassette transporter A1. Diabetes. (2002) 51:2922–8. doi: 10.2337/diabetes.51.10.2922, PMID: 12351428

[33] Uehara Y Miura S von Eckardstein A Abe S Fujii A Matsuo Y. Unsaturated fatty acids suppress the expression of the ATP-binding cassette transporter G1 (ABCG1) and ABCA1 genes via an LXR/RXR responsive element. Atherosclerosis. (2007) 191:11–21. doi: 10.1016/j.atherosclerosis.2006.04.018, PMID: 16730733

[34] Vigna GB Satta E Bernini F Boarini S Bosi C Giusto L. Flow-mediated dilation, carotid wall thickness and HDL function in subjects with hyperalphalipoproteinemia. Nutr Metab Cardiovasc Dis. (2014) 24:777–83. doi: 10.1016/j.numecd.2014.02.010, PMID: 24680225

[35] Rohatgi A Khera A Berry JD Givens EG Ayers CR Wedin KE. HDL cholesterol efflux capacity and incident cardiovascular events. N Engl J Med. (2014) 371:2383–93. doi: 10.1056/NEJMoa1409065, PMID: 25404125 PMC4308988

[36] Ishikawa T Ayaori M Uto-Kondo H Nakajima T Mutoh M Ikewaki K. High-density lipoprotein cholesterol efflux capacity as a relevant predictor of atherosclerotic coronary disease. Atherosclerosis. (2015) 242:318–22. doi: 10.1016/j.atherosclerosis.2015.06.028, PMID: 26246268

[37] Ronsein GE Vaisar T. Inflammation, remodeling, and other factors affecting HDL cholesterol efflux. Curr Opin Lipidol. (2017) 28:52–9. doi: 10.1097/MOL.0000000000000382, PMID: 27906712 PMC5567787

[38] Hernaez A Castaner O Elosua R Pinto X Estruch R Salas-Salvado J. Mediterranean diet improves high-density lipoprotein function in high-cardiovascular-risk individuals: a randomized controlled trial. Circulation. (2017) 135:633–43. doi: 10.1161/CIRCULATIONAHA.116.023712, PMID: 28193797

[39] Hernaez A Fernandez-Castillejo S Farras M Catalan U Subirana I Montes R. Olive oil polyphenols enhance high-density lipoprotein function in humans: a randomized controlled trial. Arterioscler Thromb Vasc Biol. (2014) 34:2115–9. doi: 10.1161/ATVBAHA.114.303374, PMID: 25060792

[40] Uto-Kondo H Ayaori M Ogura M Nakaya K Ito M Suzuki A. Coffee consumption enhances high-density lipoprotein-mediated cholesterol efflux in macrophages. Circ Res. (2010) 106:779–87. doi: 10.1161/CIRCRESAHA.109.206615, PMID: 20075335

[41] Zhu Y Huang X Zhang Y Wang Y Liu Y Sun R. Anthocyanin supplementation improves HDL-associated paraoxonase 1 activity and enhances cholesterol efflux capacity in subjects with hypercholesterolemia. J Clin Endocrinol Metab. (2014) 99:561–9. doi: 10.1210/jc.2013-2845, PMID: 24285687

[42] Cho KH Kim SJ Yadav D Kim JY Kim JR. Consumption of Cuban policosanol improves blood pressure and lipid profile via enhancement of HDL functionality in healthy women subjects: randomized, double-blinded, and placebo-controlled study. Oxidative Med Cell Longev. (2018) 2018:4809525–15. doi: 10.1155/2018/4809525, PMID: 29854085 PMC5944267

[43] Villard EF EI Khoury P Frisdal E Bruckert E Clement K Bonnefont-Rousselot D. Genetic determination of plasma cholesterol efflux capacity is gender-specific and independent of HDL-cholesterol levels. Arterioscler Thromb Vasc Biol. (2013) 33:822–8. doi: 10.1161/ATVBAHA.112.300979, PMID: 23372063

[44] Aicher BO Haser EK Freeman LA Carnie AV Stonik JA Wang X. Diet-induced weight loss in overweight or obese women and changes in high-density lipoprotein levels and function. Obesity (Silver Spring). (2012) 20:2057–62. doi: 10.1038/oby.2012.56, PMID: 22402736 PMC3374067

[45] Ishigami M Yamashita S Sakai N Hirano K Arai T Maruyama T. High-density lipoproteins from probucol-treated patients have increased capacity to promote cholesterol efflux from mouse peritoneal macrophages loaded with acetylated low-density lipoproteins. Eur J Clin Investig. (1997) 27:285–92. doi: 10.1046/j.1365-2362.1997.1040657.x, PMID: 9134376

[46] Ferrara A Barrett-Connor E Shan J. Total, LDL, and HDL cholesterol decrease with age in older men and women. The Rancho Bernardo Study 1984-1994. Circulation. (1997) 96:37–43. doi: 10.1161/01.cir.96.1.37 9236414

[47] Milman S Atzmon G Crandall J Barzilai N. Phenotypes and genotypes of high density lipoprotein cholesterol in exceptional longevity. Curr Vasc Pharmacol. (2014) 12:690–7. doi: 10.2174/1570161111666131219101551, PMID: 24350928 PMC4087084

[48] Lee EY Yoo JA Lim SM Cho KH. Anti-aging and tissue regeneration ability of policosanol along with lipid-lowering effect in hyperlipidemic zebrafish via enhancement of high-density lipoprotein functionality. Rejuvenation Res. (2016) 19:149–58. doi: 10.1089/rej.2015.1745, PMID: 26413884 PMC4841090

[49] Lee JY Choi HY Kang YR Chang HB Chun HS Lee MS. Effects of long-term supplementation of policosanol on blood cholesterol/glucose levels and 3-hydroxy-3-methylglutaryl coenzyme a reductase activity in a rat model fed high cholesterol diets. Food Sci Biotechnol. (2016) 25:899–904. doi: 10.1007/s10068-016-0147-y, PMID: 30263351 PMC6049138

[50] Castano G Mas R Fernandez J Lopez E Illnait J Fernandez L. Effects of policosanol on borderline to mildly elevated serum total cholesterol levels: a prospective, double-blind, placebo-controlled, parallel-group, comparative study. Curr Ther Res Clin Exp. (2003) 64:522–37. doi: 10.1016/j.curtheres.2003.09.002, PMID: 24944402 PMC4053045

[51] NCD Risk Factor Collaboration. National trends in total cholesterol obscure heterogeneous changes in HDL and non-HDL cholesterol and total-to-HDL cholesterol ratio: a pooled analysis of 458 population-based studies in Asian and Western countries. Int J Epidemiol. (2020) 49:173–92. doi: 10.1093/ije/dyz099, PMID: 31321439 PMC7245049

[52] Yokoyama S. Continuous and marked increase of Japanese HDL associates paradoxically with their nutritional shift. J Atheroscler Thromb. (2023) 30:1288. doi: 10.5551/jat.ER63894, PMID: 37532568 PMC10499448

[53] Madsen CM Varbo A Nordestgaard BG. Novel insights from human studies on the role of high-density lipoprotein in mortality and noncardiovascular disease. Arterioscler Thromb Vasc Biol. (2021) 41:128–40. doi: 10.1161/ATVBAHA.120.314050, PMID: 33232200

[54] Brewer HB Jr. Clinical review: The evolving role of HDL in the treatment of high-risk patients with cardiovascular disease. J Clin Endocrinol Metab. (2011) 96:1246–57. doi: 10.1210/jc.2010-0163, PMID: 21389140

[55] Teslovich TM Musunuru K Smith AV Edmondson AC Stylianou IM Koseki M. Biological, clinical and population relevance of 95 loci for blood lipids. Nature. (2010) 466:707–13. doi: 10.1038/nature09270, PMID: 20686565 PMC3039276

[56] Armitage J Holmes MV Preiss D. Cholesteryl ester transfer protein inhibition for preventing cardiovascular events: JACC review topic of the week. J Am Coll Cardiol. (2019) 73:477–87. doi: 10.1016/j.jacc.2018.10.072, PMID: 30704580 PMC6354546

[57] Kim JY Kim SM Kim SJ Lee EY Kim JR Cho KH. Consumption of policosanol enhances HDL functionality via CETP inhibition and reduces blood pressure and visceral fat in young and middle-aged subjects. Int J Mol Med. (2017) 39:889–99. doi: 10.3892/ijmm.2017.2907, PMID: 28259941 PMC5360427

[58] Millar JS Lassman ME Thomas T Ramakrishnan R Jumes P Dunbar RL. Effects of CETP inhibition with anacetrapib on metabolism of VLDL-TG and plasma apolipoproteins C-II, C-III, and E. J Lipid Res. (2017) 58:1214–20. doi: 10.1194/jlr.M074880 28314859 PMC5454510

[59] McLaren DG Previs SF Phair RD Stout SJ Xie D Chen Y. Evaluation of CETP activity *in vivo* under non-steady-state conditions: influence of anacetrapib on HDL-TG flux. J Lipid Res. (2016) 57:398–409. doi: 10.1194/jlr.M063842 26658238 PMC4766989

[60] van Capelleveen JC Kastelein JJ Zwinderman AH van Deventer SJ Collins HL Adelman SJ. Effects of the cholesteryl ester transfer protein inhibitor, TA-8995, on cholesterol efflux capacity and high-density lipoprotein particle subclasses. J Clin Lipidol. (2016) 10:1137–1144.e3. doi: 10.1016/j.jacl.2016.06.006, PMID: 27678430

[61] Singh SA Andraski AB Higashi H Lee LH Ramsaroop A Sacks FM. Metabolism of PLTP, CETP, and LCAT on multiple HDL sizes using the orbitrap fusion lumos. JCI Insight. (2021) 6:e143526. doi: 10.1172/jci.insight.143526, PMID: 33351780 PMC7934878

[62] Menendez R Fernandez SI Del Rio A Gonzalez RM Fraga V Amor AM. Policosanol inhibits cholesterol biosynthesis and enhances low density lipoprotein processing in cultured human fibroblasts. Biol Res. (1994) 27:199–203. PMID: 8728831

