# Supplementary material for: Cuban policosanol improves high-density lipoprotein cholesterol efflux capacity in healthy Japanese subjects
Source: Front Nutr. 2024 Jan 8;10:1297008. doi: 10.3389/fnut.2023.1297008 (PMC10800607; doi:10.3389/fnut.2023.1297008)
Supplement: Supplementary file 1 [file Data_Sheet_1.pdf]

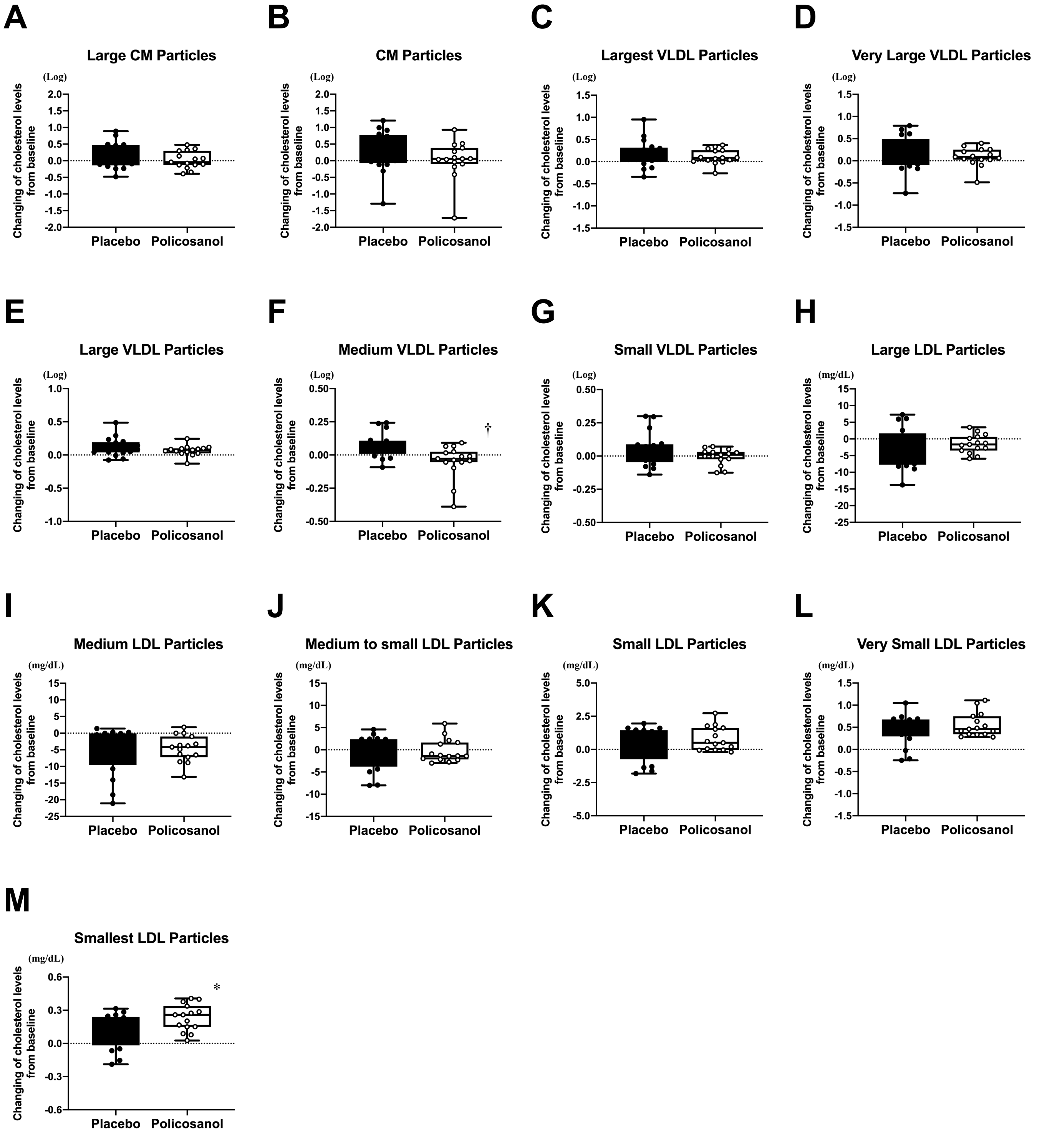

Supplementary Figure S1. Cholesterol level analysis by CM, VLDL, and LDL particle size using the HPLC method after a 12-week policosanol supplementation. The data demonstrated (**A**) large CM particles, (**B**) CM particles, (**C**) largest VLDL particles, (**D**) very large VLDL particles, (**E**) large VLDL particles, (**F**) medium VLDL particles, (**G**) small VLDL particles, (**H**) large LDL particles, (**I**) medium LDL particles, (**J**) medium to small LDL particles, (**K**) small LDL particles, (**L**) very small LDL particles, and (**M**) smallest LDL particles. Values represent the mean and minimum to maximum. Values of fractions 1–7 of cholesterol in both groups were log-transformed prior to analysis. *$p < 0.05$; †$p < 0.01$ vs. placebo. Placebo group, n = 17 and policosanol group, n = 15. Abbreviations: CM, chylomicron; VLDL, very low-density lipoprotein cholesterol; LDL, low-density lipoprotein cholesterol.

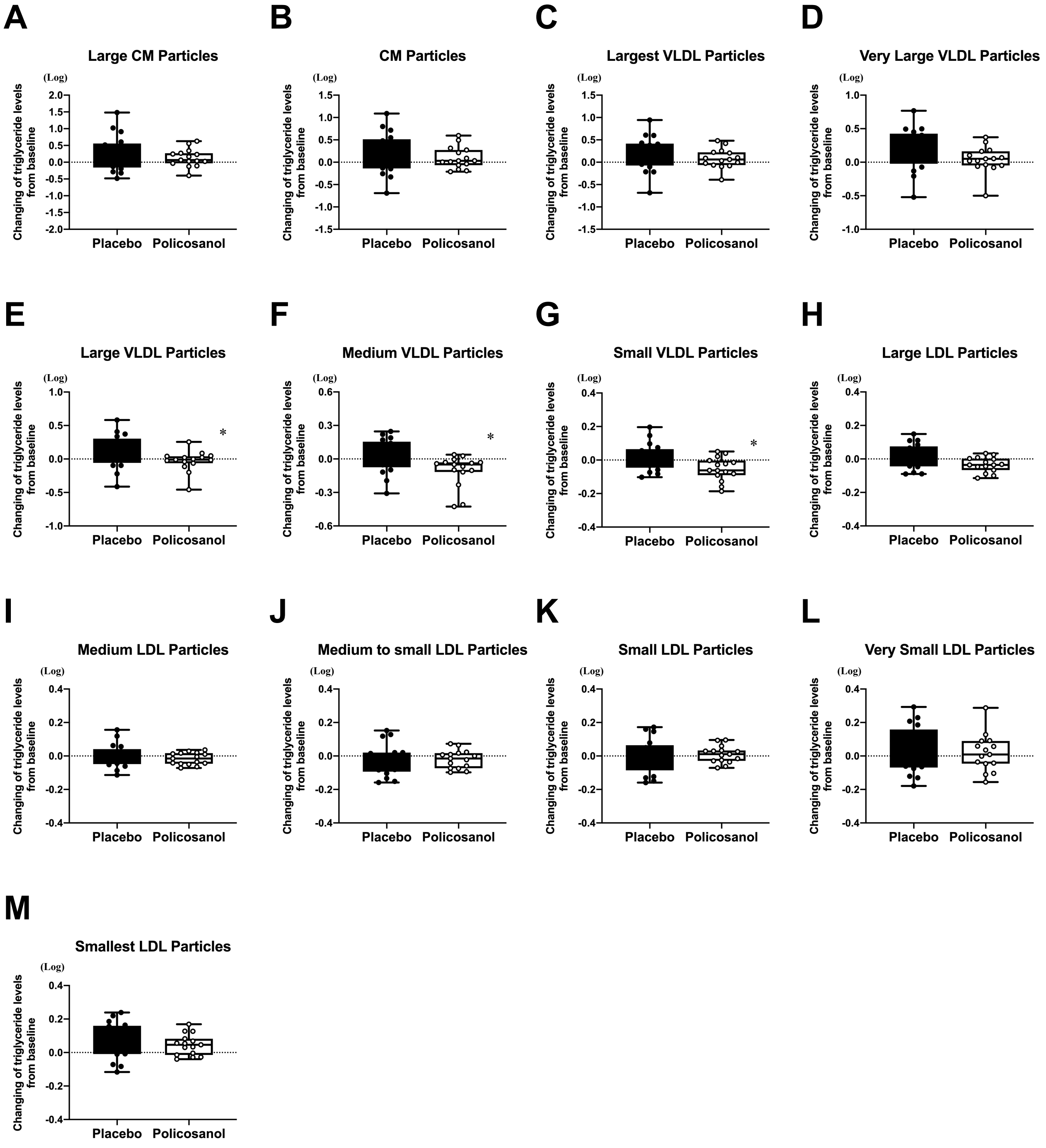

Supplementary Figure S2. Triglyceride level analysis by CM, VLDL and LDL particle size using the HPLC method after policosanol supplementation during 12 weeks. The data revealed (**A**) large CM particles, (**B**) CM particles, (**C**) largest VLDL particles, (**D**) very large VLDL particles, (**E**) large VLDL particles, (**F**) medium VLDL particles, (**G**) small VLDL particles, (**H**) large LDL particles, (**I**) medium LDL particles, (**J**) medium to small LDL particles, (**K**) small LDL particles, (**L**) very small LDL particles and (**M**) smallest LDL particles. Values represent the mean and minimum to maximum. In both groups, values of all triglyceride fractions were log-transformed before analysis. *$p < 0.05$ vs. placebo. Placebo group, n = 17 and policosanol group, n = 15. Abbreviations: CM, chylomicron; VLDL, very low-density lipoprotein cholesterol; LDL, low-density lipoprotein cholesterol.

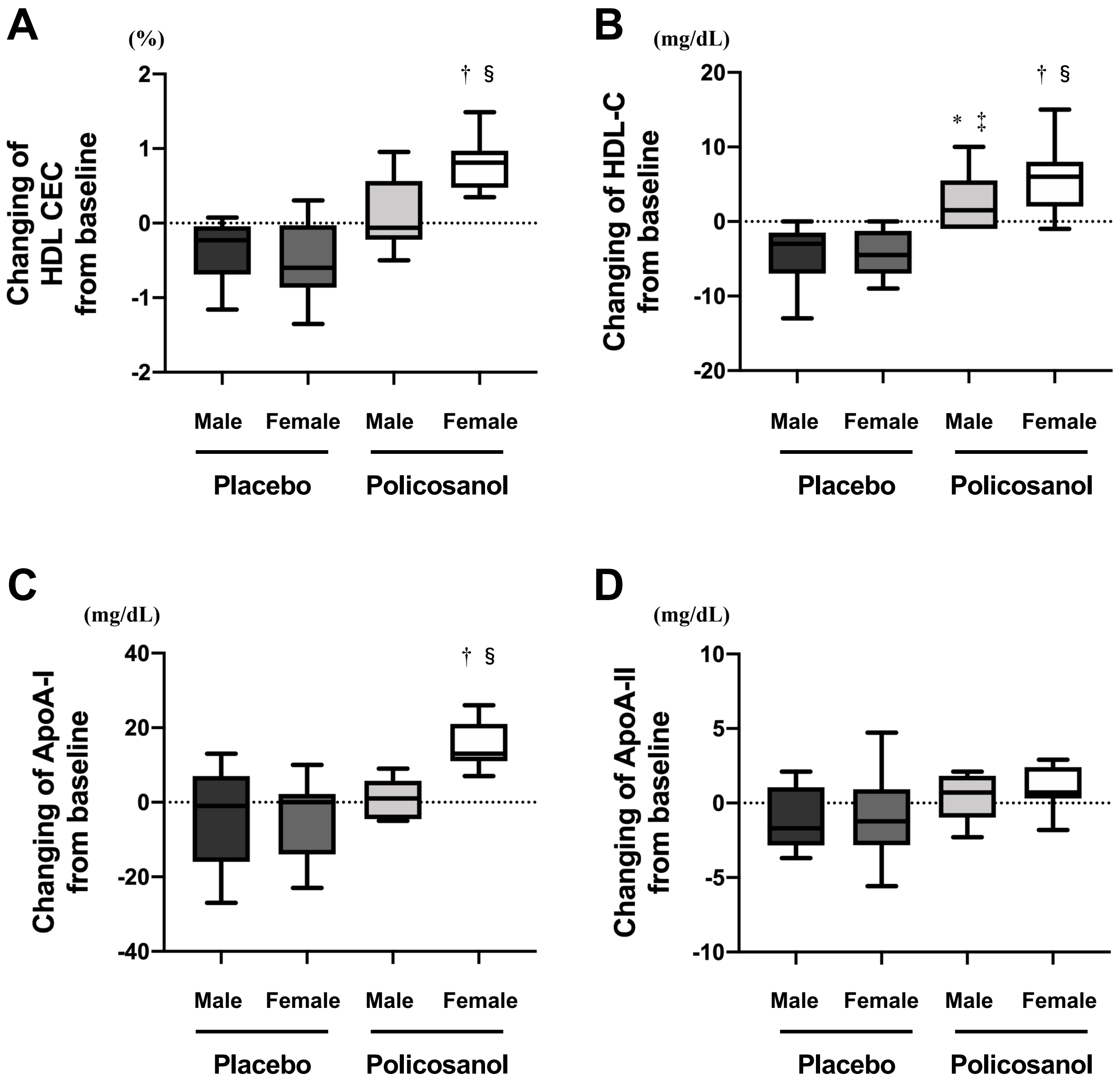

Supplementary Figure S3. Expression of policosanol effects in the four groups stratified by sexes, targeting (A) HDL CEC, (B) HDL-C, (C) ApoA-I, and (D) ApoA-II levels. Analysis was performed on the differences between the pre- and post-intervention values. Values are presented as mean and minimum to maximum. *$p < 0.05$; †$p < 0.01$ vs. male in the placebo group, ‡$p < 0.05$; §$p < 0.01$ vs. female in the placebo group. Abbreviations: HDL, high-density lipoprotein; CEC, cholesterol efflux capacity; Apo, apolipoprotein.
