# Supplementary material for: Cuban policosanol improves high-density lipoprotein cholesterol efflux capacity in healthy Japanese subjects
Source: Front Nutr. 2024 Jan 8;10:1297008. doi: 10.3389/fnut.2023.1297008 (PMC10800607; doi:10.3389/fnut.2023.1297008)
Supplement: Supplementary file 2 [file Data_Sheet_2.pdf]

Supplementary Table S1. Baseline characteristics of the four groups categorized according to sexes. Values represent mean ± SD or median with interquartile range. [1]Triglyceride and apolipoprotein B-48 in both groups were log-transformed before analysis. Abbreviations: HDL, high-density lipoprotein; LDL, low-density lipoprotein cholesterol.

	Placebo		Policosanol	
	Male (n = 9)	Female (n = 8)	Male (n = 8)	Female (n = 7)
HDL-cholesterol, mg/dL	62.6 ± 10.0	70.1 ± 8.7	60.5 ± 8.2	67.4 ± 14.0
HDL cholesterol efflux capacity, %	15.6 ± 0.9	16.2 ± 0.8	15.7 ± 0.4	15.4 ± 1.0
Total cholesterol, mg/dL	213.8 ± 18.8	231.4 ± 19.9	216.8 ± 15.7	219.1 ± 14.7
LDL-cholesterol, mg/dL	130.1 ± 12.6	137.1 ± 18.3	127.9 ± 12.4	125.0 ± 9.2
Triglycerides, mg/dL[1]	74.0 [54.0-90.0]	70.0 [53.3-82.3]	90.0 [68.8-106.8]	81.0 [54.0-125.0]
Apolipoprotein AI, mg/dL	167.6 ± 20.4	182.0 ± 15.6	170.5 ± 18.6	178.0 ± 20.4
Apolipoprotein AII, mg/dL	35.8 ± 5.0	35.2 ± 2.9	34.9 ± 3.2	32.6 ± 3.0
Apolipoprotein B100, mg/dL	82.3 ± 14.6	78.7 ± 9.2	92.4 ± 15.2	82.9 ± 18.0
Apolipoprotein B48, mg/dL[1]	0.38 [0.24-0.44]	0.42 [0.30-0.69]	0.32 [0.18-0.62]	1.11 [0.46-1.15]
